# Dilution with three different solutions: plasmatic effects and quantity and quality of urinary output

**DOI:** 10.1186/cc9506

**Published:** 2011-03-11

**Authors:** T Langer, E Carlesso, A Protti, M Monti, L Zani, G Iapichino, B Comini, D Andreis, C Sparacino, D Dondossola, L Gattinoni

**Affiliations:** 1 Università degli Studi di Milano, Milan, Italy; 2 Centro Ricerche Chirurgiche Precliniche, Università degli Studi, Milan, Italy

## Introduction

Crystalloids have different electrolyte composition and therefore different strong ion differences (SIDinf). The aim of the study was to investigate the response of the kidney to plasmatic acid-base changes induced by dilution with three crystalloid solutions at different SID.

## Methods

Six pigs (22 ± 4 kg) were anesthetized and mechanically ventilated. The respiratory rate was adjusted to maintain pCO_2 _constant. A urinary catheter was placed and connected to a urinary analyzer (Orvim, Paderno Dugnano, Italy) [1]. Pigs were randomly assigned to a sequence of dilutions (10% of body weight in 2 hours, followed by 4 hours of washout period) with the following three fluids: normal saline (NS), SID = 0, [Na] = 154, [Cl] = 154; lactated Ringer's (LR), SID = 29, [Na] = 132, [Cl] = 112; and polysaline RIII (RIII), SID = 55, [Na] = 140, [Cl] = 103. Blood gases and electrolytes as well as urinary pH (pHu), urinary electrolytes and urinary output (UO) were recorded at baseline and at the end of each dilution. Plasmatic SID was defined as [Na] + [K] + 2[Ca] - [Cl] - [Lac]. Variations (d) were defined as baseline - 2-hour value.

## Results

Plasmatic changes are consistent with previous *in vitro *studies [2]. dSID was mainly due to d[Cl]: 9.0 ± 1.7 for NS, no change for LR, -1.5 ± 1.6 for RIII. Of note, while no difference was yet observed for urinary electrolytes and UO, pHu significantly differed between the three solutions. See Table 1.

**Table 1:** 

	NS	LR	RIII
dpHa	-0.08 ± 0.02	-0.00 ± 0.03*	0.04 ± 0.03*^#^
dSID (mEq/l)	-4.56 ± 1.36	0.21 ± 1.41*	3.56 ± 1.95*^#^
dpHu	-0.16 ± 0.47	0.23 ± 0.44	1.00 ± 0.75*^#^
UO (ml)	639 ± 235	768 ± 172	896 ± 293

Data presented as mean ± standard deviation. *P < 0.05 vs. NS. ^#^*P *< 0.05 vs. LR. One-way ANOVA RM.

## Conclusions

The quality of infused fluids affects greatly the acid-base and electrolyte equilibrium of plasma. This in turn alters the quality of urine (pHu).
